# Association between Cluster of Lifestyle Behaviors and HOMA-IR among Adolescents: ABCD Growth Study

**DOI:** 10.3390/medicina54060096

**Published:** 2018-12-01

**Authors:** André Oliveira Werneck, Ricardo Ribeiro Agostinete, Suziane Ungari Cayres, Jacqueline Bexiga Urban, Andréa Wigna, Lucas Gabriel de Moraes Chagas, Wesley Torres, Rômulo Araújo Fernandes

**Affiliations:** Laboratory of InVestigation in Exercise—LIVE, Department of Physical Education, Sao Paulo State University (UNESP), Presidente Prudente, Sao Paulo 19060-900, Brazil; ricardoagostinete@gmail.com (R.R.A.); suziungari@yahoo.com.br (S.U.C.); jac_bexiga@yahoo.com.br (J.B.U.); aw.wigna@gmail.com (A.W.); lucas.charaes@gmail.com (L.G.d.M.C.); wesleytorres_wt@yahoo.com.br (W.T.); romulo.a.fernandes@unesp.br (R.A.F.)

**Keywords:** insulin resistance, health risk behaviors, adiposity, youth

## Abstract

*Objective:* To analyze the association of potential risk factors to health with body fatness and insulin resistance. Baseline measures of the ongoing longitudinal Analysis of Behaviors of Children During (ABCD) Growth Study. *Materials and Methods:* The sample was composed of 280 adolescents of both sexes (198 boys and 82 girls) aged from 10 to 18 years. Four risk factors were considered, as follows: no sports practice, skipping breakfast, poor sleep quality, and TV viewing. The outcomes considered were insulin resistance (HOMA-IR) and body fatness (densitometer scanner). Age, sex, maturity offset, and ethnicity were treated as covariates. *Results:* No sports practice and skipping breakfast were associated with higher body fatness (Sports practice: Wald: 8.786; *p* = 0.003. Breakfast: Wald: 9.364; *p* = 0.002). Poor sleep quality was related to a greater HOMA-IR index (Wald: 6.013; *p* = 0.014). Adolescents with ≥3 risk factors presented a higher risk of high HOMA-IR (OR = 4.89 (95%CI: 1.61 to 14.84)) than their counterparts with no risk factors. *Conclusion:* Lifestyle risk factors seem relevant to affect obesity and insulin resistance, while the aggregation of these risk factors affects insulin resistance, independent of adiposity.

## 1. Introduction

Homeostatic Model Assessment of Insulin Resistance (HOMA-IR), derived from an equation with fasting glucose and insulin, is an important technique for the diagnosis and surveillance of insulin resistance, being a marker of type 2 diabetes mellitus [1,2]. Type 2 diabetes mellitus is an important public health problem, affecting 422 million people in 2014, and increases the risk of developing cardiovascular diseases such as hypertension and dyslipidemia [3,4]. Since early life (childhood and adolescence), IR has been related to different health outcomes, such as higher triglycerides, higher total cholesterol, lower high-density lipoprotein (HDL), and changes in blood pressure [5].

Insulin resistance is strongly affected by body fatness [6]. It is known that adipose tissue is a metabolically active tissue responsible for the secretion of protein and non-protein factors [7,8,9]. Although all mechanisms underlying the link between obesity and IR are not entirely clear, it is known that hormones and cytokines such as resistin, tumor necrosis factor (TNF-α), and interleukin-6 (IL-6) secreted by adipocytes unbalance the action of insulin [7,8,9]. Obesity is an outcome of multifactorial origin [10] being affected by lifestyle factors that also affect IR [11,12] and, thus the role of obesity in the relationship between insulin resistance and these lifestyle behaviors remains unclear.

Moreover, the current literature seeks to understand the effects of different lifestyle behaviors on obesity, and metabolic and cardiovascular health, such as alcohol consumption, tobacco, practice of physical exercise, and dietary patterns [13,14,15] as a study developed by Garoufi et al. (2017) [14], who related overweight/obesity with behavioral habits (smoking, physical activity, screen time and dietary habits) and its association with blood pressure and lipid profile and concluded that behavioral habits affect obesity and cardiovascular risk during adolescence. Other recent study developed by Silva et al. (2017) [15] found that adolescents who were active during childhood until adolescence had better cardiorespiratory fitness, as well as a lower risk of developing hypertension and high metabolic risk. However, there are other not so commonly investigated lifestyle behaviors (e.g., sports participation, skipping breakfast, poor sleep quality, and television viewing (TV viewing)), which can affect health outcomes even during adolescence, including obesity and insulin resistance.

For instance, sport participation, which is a domain of physical activity, is the most relevant manifestation of physical exercise mainly among adolescents, but little is known about its impact on health [15,16], as when physical activity is assessed among adolescents the burden of sports participation is seldom considered. Skipping breakfast and TV viewing (one domain of sedentary behavior) are common behaviors among adolescents, which are linked to body weight gain, and lipid and glucose abnormalities [17,18]. Moreover, sleep quality is a fundamental aspect of human health in all periods of life [19], but the investigation of poor sleep quality and its impacts on adolescent health seems to be a neglected aspect in the literature.

Thus, the aim of this study is to analyze the association between lifestyle behaviors on insulin resistance, as well as to identify whether adiposity affects this association. Based in previous findings, we hypotheses is that cluster of lifestyle behaviors are associated with insulin resistance and adiposity can affect this association.

## 2. Materials and Methods

### 2.1. Sampling

The present study is part of the ongoing longitudinal study “Analysis of Behaviors of Children During Growth” (ABCD Growth Study), which is designed to identify the impact of sport participation and other lifestyle behaviors on different health aspects of adolescents. Since 2017, the ABCD Growth Study has been ongoing in the city of Presidente Prudente in the state of São Paulo, Brazil. The Laboratory of InVestigation in Exercise (LIVE) linked to the Department of Physical Education of the São Paulo State University (UNESP) through its researchers is responsible for the ABCD Growth Study. The ethics committee of São Paulo State University (UNESP) approved the study (process number 1.677.938/2016) and parents/legal guardians and the adolescents themselves signed a written consent form before taking part in any assessment.

Researchers contacted adolescents aged 11–18 years at schools (public and private) and sports clubs of the metropolitan region of the city, after previous authorization from principals and coaches, respectively. Adolescents were contacted to explain all the objectives and inclusion criteria of the study, as well as which ethical forms were delivered to request parental/guardian authorization. Inclusion criteria were adopted as follow: (1) 11–18 years of age; (2) parents’ consent form signed; (3) if athletes, at least one year of training experience; if schoolchildren, at least one year without practicing any organized sport. Adolescents who agreed to participate and brought back the ethical forms properly signed by their parents were included in the baseline measures of the ABCD Growth Study.

After the fieldwork, 285 adolescents registered in 11 schools/sports clubs were involved in the baseline measures of the ABCD Growth Study. The baseline measurements were developed in two steps: (1) Questionnaires and analysis of body composition in university laboratories and (2) Blood samples collected in a private laboratory collaborating in the study. However, 27 adolescents, even providing the informing consent, had missing data in some variables (*n* = 5 in body composition (fat mass) and *n* = 22 in blood sample (HOMA-IR)) and were excluded from this study, leading to a final sample size of 280 adolescents for fat mass variable and 263 for HOMA-IR of both sexes.

### 2.2. HOMA-IR

Blood samples were collected after 10 h fasting by a trained nurse in a private laboratory (glucose and insulin were assessed). Insulin levels were analyzed by the chemiluminescence method using a microparticle immunoassay kit (brand ABBOTT), processed in a biochemical Autohumalyzer (brand ARCHITECT, model i2000) obtained from the ABBOTT Diagnostics company, Green Oaks, Illinois, USA. The HOMA-IR (Homeostatic Model Assessment-Insulin resistance) was calculated using (fasting insulin (mcUI/mL) × fasting glucose (mg/dL))/405 [20]. HOMA-IR ≥ 2.0 was classified as high HOMA-IR [20,21,22].

### 2.3. Body Fatness

Fat mass (in percentage values (%)) was assessed using dual-energy X-ray absorptiometry (Lunar DPX-NT scanner; General Electric Healthcare, Little Chalfont, Buckinghamshire, UK) with GE Medical System Lunar software (version 4.7). This software (Lunar DPX) provide accurate and precise results of bone and tissue composition (lean soft tissue and fat mass) and the measurements are fast and non-invasive. The scanner quality was tested by a trained researcher before each day of measurement, following the manufacturer’s recommendations, and all scans were carried out at the university laboratory in a temperature-controlled room by the same researcher and following the International Society of Clinical Densitometry guidelines [23]. The participants wore light clothing, without shoes and remained in the supine position on the machine (approximately 15 min). The precision of the machine in terms of coefficient of variation was 0.66% (*n* = 30 subjects not involved in this study).

### 2.4. Cluster of Lifestyle Behaviors

Lifestyle behaviors were assessed through a face-to-face interview, as follows: (1) No sport participation: the absence of sport participation was performed considering the sample selection, in which, were selected adolescents from sports clubs (considered as the group with sports practice) and adolescents from elementary and high schools, which did not practice sports (considered as the no sports participation group). (2) Skipping breakfast: the adolescent reported, considering a normal week, the number of days that they consumed breakfast (ranging from zero to seven days). Adolescents who reported their breakfast intake <7 days/week were characterized as having the habit of “skipping breakfast”. (3) Sleep quality: Quality of sleep was assessed using the Mini-Sleep Questionnaire (MSQ), translated to Brazilian Portuguese [24]. The questionnaire is composed of 10 questions with 7 possible answers (ranging from 0 to 7; never = 1, very rarely = 2, rarely = 3, sometimes = 4, often = 5, very often = 6, and always = 7) and the sum of the answers generates a numerical score. Adolescents who presented a score ≥25 points were characterized as “poor sleep quality” [24]. (4) TV viewing: Time spent in front of a TV was assessed by one question in the leisure-time section of the Baecke questionnaire [25]: “During leisure time I watch television” (possible responses: never, seldom, sometimes, often, and very often). Adolescents that responded “very often” in the Likert scale were characterized in “High TV viewing”.

Lifestyle behaviors were treated in two ways, separately and clustered together (variables ranging from zero (no lifestyle behavior) to four (simultaneous presence of skipping breakfast, poor sleep quality, no sport participation, and high TV viewing)). In terms of frequency, the cluster variable was divided as follow: None (*n* = 35, 12.3%), one lifestyle behavior (*n* = 98, 34.4%), two lifestyle behaviors (*n* = 102, 35.8%), three lifestyle behaviors (*n* = 46, 16.1%), and four lifestyle behaviors (*n* = 4, 1.4%). For statistical purposes, categories three and four of lifestyle behaviors were merged into one category (*n* = 50, 17.5%).

### 2.5. Covariates

Sex, chronological age, ethnicity, and somatic maturation were treated as covariates. Body weight (kg) was measured using a digital scale (Filizzola PL 150; Filizzola Ltd., São Paulo, Brazil) and height (cm) using a stadiometer with a precision of 0.1 cm. Both measures were collected using standard protocols and used to calculate the Body Mass Index (BMI). In addition, measurements of the sitting height and length of the legs were performed to calculate the maturity deviation, which denotes the time (years) from/to the peak of height velocity (PHV) proposed by Mirwald (2002) [26], an important maturational event.

### 2.6. Statistical Analyses

The descriptive analyses were composed of mean and standard deviation. General estimating equations (GEE), including the analysis of Wald test, were used to analyze the association between lifestyle behaviors, body fatness, and HOMA-IR (isolated and cluster) adjusted by sex, chronological age, maturity offset, ethnicity, and body fatness (for HOMA analysis). The logistic regression analyzed the association between the lifestyle behaviors cluster (no sport participation, high TV viewing, skipping breakfast, and poor sleep quality) and HOMA-IR in different models. All analyzes were performed in software SPSS (version 24.0) and the significance value was previously set at *p*-value < 0.05.

## 3. Results

General characteristics of the sample, stratified according to sex, are presented in Table 1. Girls presented greater maturity offset and body fatness (*p*-value < 0.001), while all lifestyle behaviors, even presenting a considerable prevalence of ≥3 lifestyles behavior risk in both sexes (boys: 18.2% vs. Girls: 17.1%), were similar between boys and girls even when considered as a cluster variable (*p*-value = 0.351).

Table 2 presents GEE of the association between isolated lifestyle behaviors and outcomes (body fatness and HOMA-IR), in which results were adjusted by sex, chronological age, maturity offset, ethnicity, and BMI (for HOMA-IR analysis). Adolescents who were not engaged in sport participation as well as frequently skipping breakfast presented higher body fatness (Sport participation: Wald: 8.786; *p*-value = 0.003; Breakfast: Wald: 9.364; *p*-value = 0.002). Moreover, poor sleep quality was associated with a higher HOMA-IR index (Wald: 6.013; *p*-value = 0.014).

**Figure 1:**
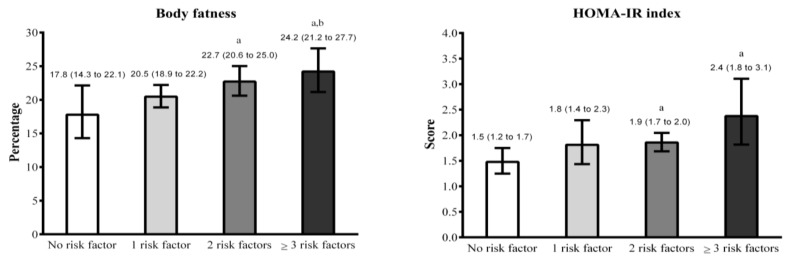
General estimating equations of the association between lifestyle behavior clusters (no sport participation, high TV-viewing, skipping breakfast, and poor sleep quality). Note. Values are presented as means and 95% standard deviations. Models are adjusted by sex, chronological age, maturity offset, ethnicity, and body fatness (for HOMA-IR analysis). a = significant difference vs. no lifestyle behavior group (*p* < 0.05). b = significant difference vs. 1 lifestyle behavior group (*p* < 0.05).

After the first set of analyses, we created a cluster indicator of lifestyle behaviors, with four categories (no lifestyle behavior, 1 lifestyle behavior, 2 lifestyle behaviors, ≥3 lifestyle behaviors). Therefore, GEE of the association between cluster of lifestyle behaviors, body fatness, and HOMA-IR are presented in Figure 1. Adolescents with no lifestyle behavior presented lower body fatness than their counterparts with 2 lifestyle behaviors (*p*-value = 0.031) and ≥3 lifestyle behaviors (*p*-value = 0.014), while the group with 1 lifestyle behavior presented lower body fatness than the group with ≥3 lifestyle behaviors (*p*-value = 0.045). Similarly, adolescents with no lifestyle behaviors presented a lower HOMA-IR index than their peers with 2 lifestyle behaviors (*p*-value = 0.003) and ≥3 lifestyle behaviors (*p*-value = 0.006).

Regarding categorical HOMA-IR, the prevalence of an elevated HOMA-IR index was higher among subjects with no lifestyle behaviors: 17.6% (CI 95%: 7.8% to 35.1%) vs. 1 lifestyle behavior: 29.2% (CI 95%: 20.8% to 39.2%), 2 lifestyle behaviors: 38.0% (CI 95%: 28.9% to 48.0%), and ≥3 lifestyle behaviors: 52.0% (CI 95%: 37.9% to 65.8%) (*p*-trend: 0.005). Logistic regression analyses of the association between lifestyle behavior cluster and an elevated HOMA-IR index are presented in Table 3. After the adjustment by sex, age, maturity offset, and ethnicity (model 2), clusters of two (*p*-value = 0.020) and three (*p*-value = 0.001) lifestyle behaviors were more likely to present elevated HOMA-IR when compared to subjects with no lifestyle behaviors. However, after the adjustment by body fatness (Model 3), only presenting 3 or more lifestyle behaviors was considered a risk factor for elevated HOMA-IR (389% more likely; *p*-value = 0.005).

## 4. Discussion

Although the association between “traditional” lifestyle behaviors (e.g., dietary patterns and physical activity) and HOMA-IR is widely explored in the literature [27,28,29], we tried to analyze the association between not so conventional lifestyle behaviors and HOMA-IR. Our main findings identified that these lifestyle behaviors can affect adiposity levels among adolescents, added to which, when they are considered as cluster variables, they are able to affect the insulin resistance score among adolescents.

In this study, no sport participation and skipping breakfast were associated with higher body fatness. Firstly, it was expected that adolescents engaged in sports would present lower body fatness as sports participation usually requires performance of physical exercises at higher intensity, leading to the athlete adolescents presenting lower body adiposity than their peers [30]. Similarly, skipping breakfast was associated with higher body fatness in our study, which is also consistent with previous findings in adolescents [31,32], possibly occurring through appetite regulation mechanisms [33]. Another recent study that corroborates these findings about breakfast, was developed by Cayres et al. [34]. The authors analyzed the longitudinal relationship between breakfast intake and obesity and the mediating effect of physical activity and observed that adolescents who ate breakfast regularly, presented lower body fat independently of physical activity.

Moreover, poor sleep quality was the only lifestyle behavior associated with HOMA-IR. These findings are somewhat in agreement with previous studies that found an association between poor sleep quality/sleep deprivation and insulin resistance, especially among obese adolescents [35,36,37]. A possible mechanism underlying this association is related to an imbalance in autonomic modulation, given the fact that poor sleep quality may affect autonomic function through a decrease in parasympathetic modulation [38,39], and is also associated with impaired tissue response via insulin resistance [40]. Moreover, a poorer sleep quality is also associated with an elevation of stress levels as well as the regulation of hypothalamic-pituitary-adrenal, which can cause a neuroendocrine disorder, which in turn can cause a deregulation in the glucose-insulin metabolism [41,42].

Apparently, the main contribution of our study was to identify that aggregation of these lifestyle behaviors was related to worse body composition and metabolic health. Moreover, even after the adjustment by body fatness, adolescents that presented ≥2 lifestyle behaviors showed higher HOMA-IR levels. Taking into account qualitative analyses, adolescents who presented three or more lifestyle behaviors were more likely to present elevated HOMA-IR than adolescents with no lifestyle behavior, emphasizing the importance of the presence of none of these unhealthy lifestyle behaviors.

The non-adiposity dependent association observed between these lifestyle behaviors and HOMA-IR seems interesting due to the central role exerted by adiposity in the development of insulin resistance. Body fatness could be a possible mediator (but only partially) of sport participation and skipping breakfast in the association with HOMA-IR, given the fact that the association of both with body fatness is closely related to HOMA-IR [43]. The associations between body fatness and HOMA-IR have been justified in the scientific literature by the fact that adipose tissue is an endocrine organ and secretes hormones as well as proinflammatory cytokines such as resistin, TNF-α, and IL-6, among others [44]. Resistin is an adipocytokine that, although controversial, has been considered as the main variable explaining the relationship between obesity and IR [7,44]. The release of resistin is positively related to proinflammatory cytokines, which in specific target cells affect different tissues such as IL-6 and TNF, among others. Specifically regarding IL-6 and TNF-α [45], both are responsible for stimulation of the IκB kinase-β (IKK-β)/nuclear factor-κB (NF-κB) and the c-Jun aminoterminal kinase (JNK) through specific receptors, elevating inflammation, impairing insulin signaling [46], besides the downregulation of glucose transporters (GLUT-4) causing insulin resistance in the long run [47].

None of these four lifestyle behaviors were significantly associated with each other (data not shown), denoting an interesting independence among them, mainly because the cluster of these lifestyle behaviors seems harmful to adolescent health. As a result, parents should focus effort on combating these four lifestyle behaviors separately, which means, for example, trying to decrease the time spent watching TV not expecting an improvement in sport participation engagement as a response. In this sense, our results lead to clear practical implications, given that the avoid of health risk behaviors should be a protection for the development of body adiposity and insulin resistance. Interventions should have broader spectrums aiming to reduce several health risk behaviors.

The present study has some limitations. In terms of design, the sampling process was not randomized, and the cross-sectional design does not offer support to causality statements. Moreover, our sample size did not allow more robust statistical procedures such as modeling possible mediators or even splitting the analyses by sex. Self-report of sleep quality and TV-viewing is prone to bias, firstly because TV-viewing represent just one domain of sedentary behavior and due the access through a Likert scale. Finally, the reasons related to “skipping breakfast” or which physiological aspects of sleep presented difficulties would better explain these metabolic alterations.

## 5. Conclusions

In conclusion, we found that the clustering of lifestyle behaviors (risk) was associated with higher HOMA-IR, independent of adiposity. In this sense, the cluster of two risk behaviors (among no sports participation, high TV-viewing, skipping breakfast and poor sleep quality) is a threshold for the increase of insulin resistance. Future studies should focus on longitudinal associations between non-traditional lifestyle behaviors and insulin resistance among adolescents.

## Figures and Tables

**Table 1:** Characteristics of the sample stratified according to sex (*n* = 280).

	Boys (*n* = 198)	Girls (*n* = 82)	
Variables	Mean ± SD	Mean ± SD	*p*-Value
Continuous variables			
Chronological age (years)	14.7 ± 2.0	14.9 ± 2.1	0.451
Maturity offset (years)	0.94 ± 1.56	2.17 ± 1.34	<0.001
BMI (kg/m^2^)	21.3 ± 4.1	20.4 ± 3.4	0.070
Body fatness (%)	19.4 ± 11.1	29.0 ± 9.4	<0.001
HOMA-IR (score)	1.97 ± 1.87	1.89 ± 1.15	0.547
Categorical variables			
No sports practice (%)	57.3 (50.3 to 64.1)	53.7 (42.6 to 64.3)	0.548
High TV viewing (%)	14.7 (10.4 to 20.4)	22.0 (14.2 to 32.4)	0.142
Skipping breakfast (%)	45.2 (38.3 to 52.2)	35.4 (25.6 to 46.5)	0.121
Poor sleep quality (%)	44.2 (37.3 to 51.2)	47.6 (36.8 to 58.5)	0.580
Lifestyle behavior clusters			0.351
No lifestyle behavior (%)	13.6 (9.5 to 19.2)	8.5 (4.1 to 17.1)	
1 lifestyle behavior (%)	31.3 (25.2 to 38.2)	41.5 (31.1 to 52.6)	
2 lifestyle behaviors (%)	36.9 (30.4 to 43.9)	32.9 (23.5 to 44.0)	
≥3 lifestyle behaviors (%)	18.2 (13.4 to 24.2)	17.1 (10.3 to 27.0)	

BMI = body mass index; HOMA-IR = Homeostatic Model Assessment of Insulin Resistance. Note. Values are presented as means and standard deviations (SD) as well as frequencies and 95% confidence intervals.

**Table 2:** General estimating equations of the association between isolated lifestyle behaviors, body fatness, and the HOMA-IR index.

Prevalence of Lifestyle Behavior				
	No	Yes		
	Mean (95%CI)	Mean (95%CI)	Wald	*p*-Value
**Sport participation**				
Body fatness (%)	24.33 (22.07 to 26.83)	20.06 (18.63 to 21.61)	8.786	**0.003**
HOMA-IR _(score)_	1.82 (1.47 to 2.24)	1.93 (1.71 to 2.17)	0.189	0.663
**High TV-viewing**				
Body fatness (%)	21.83 (20.52 to 23.24)	20.95 (18.02 to 24.36)	0.242	0.623
HOMA-IR _(score)_	1.87 (1.67 to 2.11)	1.90 (1.67 to 2.15)	0.024	0.877
**Skipping breakfast**				
Body fatness (%)	19.90 (18.51 to 21.39)	23.93 (21.85 to 26.22)	9.364	**0.002**
HOMA-IR _(score)_	1.82 (1.59 to 2.09)	1.96 (1.70 to 2.27)	0.618	0.432
**Poor sleep quality**				
Body fatness (%)	21.58 (19.86 to 23.45)	21.63 (20.03 to 23.37)	0.002	0.968
HOMA-IR _(score)_	1.65 (1.54 to 1.78)	2.16 (1.79 to 2.61)	6.577	**0.010**

HOMA-IR = Homeostatic Model Assessment of Insulin Resistance. Note. Adjusted by sex, chronological age, maturity offset, ethnicity, and body fatness (for HOMA-IR analysis).

**Table 3:** Logistic regression of the association between lifestyle behavior clusters (no sports practice, high TV-viewing, skipping breakfast and poor sleep quality) and elevated HOMA-IR.

	B	OR	95%CI	*p*-Value
**Model 1**				
No lifestyle behavior	--	1.00	-	-
1 lifestyle behavior	0.68	1.97	0.73 to 5.30	0.181
2 lifestyle behaviors	1.08	2.94	1.11 to 7.78	0.030
≥3 lifestyle behaviors	1.65	5.20	1.83 to 14.8	0.002
**Model 2**				
No lifestyle behavior	-	1.00	-	-
1 lifestyle behavior	0.78	2.19	0.79 to 6.03	0.131
2 lifestyle behaviors	1.19	3.29	1.21 to 8.93	0.020
≥3 lifestyle behaviors	1.81	6.12	2.07 to 18.11	0.001
**Model 3**				
No lifestyle behavior	-	1.00	-	-
1 lifestyle behavior	0.70	2.02	0.71 to 5.70	0.185
2 lifestyle behaviors	1.01	2.74	0.99 to 7.65	0.053
≥3 lifestyle behaviors	1.59	4.89	1.61 to 14.84	0.005

Note. Model 1: adjusted by sex and chronological age. Model 2: Model 1 + maturity offset and ethnicity. Model 3: Model 2 + body fatness.
